# Atrial fibrillation and cancer: prevalence and relative risk from a nationwide study

**DOI:** 10.1016/j.rpth.2022.100026

**Published:** 2022-12-23

**Authors:** Cihan Ay, Ella Grilz, Stephan Nopp, Florian Moik, Oliver Königsbrügge, Peter Klimek, Stefan Thurner, Florian Posch, Ingrid Pabinger

**Affiliations:** 1 Department of Medicine I, Medical University of Vienna, Vienna, Austria; 2 Department of Internal Medicine, Medical University of Graz, Graz, Austria; 3 Section for Science of Complex Systems, CEMSIIS, Medical University of Vienna, Vienna, Austria; 4 Complexity Science Hub Vienna, Vienna, Austria; 5 Santa Fe Institute, Santa Fe, New Mexico, USA

**Keywords:** atrial fibrillation, cancer, nationwide study, neoplasms, prevalence

## Abstract

**Background:**

Atrial fibrillation (AF) is an increasingly recognized codiagnosis in patients with cancer.

**Objectives:**

This study aimed to provide a robust and contemporary estimate on the coprevalence and relative risk of AF in patients with cancer.

**Methods:**

We conducted a nationwide analysis, utilizing diagnosis codes from the Austrian Association of Social Security Providers dataset. Estimates of the coprevalence of cancer and AF and the relative risk of AF in patients with cancer compared with individuals without cancer were obtained as point prevalences with binomial exact confidence intervals and summarized across age groups and cancer types with random-effects models.

**Results:**

Overall, 8,306,244 persons were included in the present analysis, of whom 158,675 (prevalence estimate, 1.91%; 95% CI, 1.90-1.92) had a cancer diagnosis code and 112,827 (1.36%; 95% CI, 1.35-1.36) an AF diagnosis code, respectively. The prevalence estimate for AF in patients with cancer was 9.77% (95% CI, 9.63-9.92) and 1.19% (95% CI, 1.19-1.20) in the noncancer population. Conversely, 13.74% (95% CI, 13.54-13.94) of patients with AF had a concurrent cancer diagnosis. The corresponding age-stratified random-effects relative risk ratio for AF in patients with cancer compared with no cancer diagnosis was 10.45 (95% CI, 7.47-14.62). The strongest associations between cancer and AF were observed in younger persons and patients with hematologic malignancies.

**Conclusion:**

Cancer and AF have a substantial coprevalence in the population. This finding corroborates the concept that cancer and AF have common risk factors and pathophysiology.

## Essentials

• Atrial fibrillation (AF) and cancer are frequent coprevalent diseases.
• This nationwide study aimed to investigate the interrelation between AF and cancer.
• The prevalence estimate for AF in patients with cancer was 9.77%.
• Treating physicians should be aware of the eightfold increased risk of AF in patients with cancer.

## Graphical abstract

**Figure:**
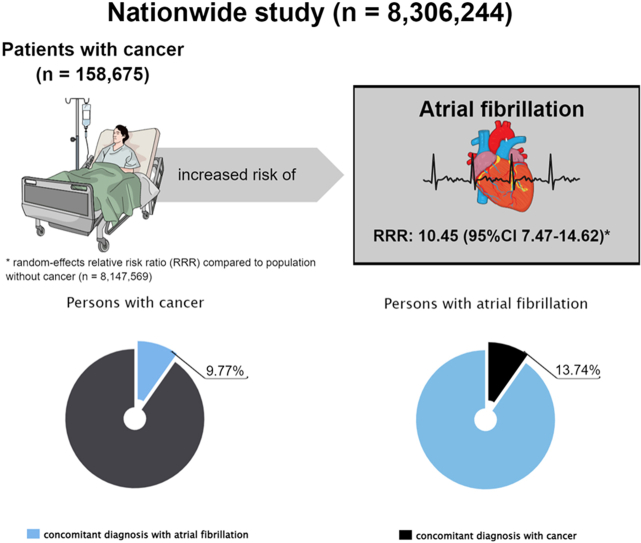

## 1 Introduction

The global cancer burden is on the rise because of the aging population. [1,2] Advances in diagnostic and therapeutic management of patients with cancer have led to improved survival and thereby to a gradual transformation of cancer toward becoming a chronic disease. [3,4] Given the increasing burden of cancer because of increased life expectancy and longer survival after cancer diagnosis, the coprevalence with secondary contributors to morbidity and mortality in patients with cancer is becoming ever more relevant today.

Atrial fibrillation (AF) represents the most common type of cardiac arrhythmia, and its prevalence increases with age. [5,6] Thus, in our aging society, the overall burden of AF is expected to rise significantly in the future. [7] AF leads to an enhanced risk of heart failure, stroke, cardiovascular death, and anticoagulation-related bleeding and thereby represents a major contributor to morbidity and mortality. [8] Because of the parallel increase of cancer and AF with age, a rising coprevalence of these two conditions is anticipated. [1,5,6] This coprevalence is enhanced by additional shared predisposing risk factors, including comorbidities, obesity, smoking, and alcohol abuse. [9,10] In addition, several factors have been suggested to be causally involved in the pathophysiology of AF in patients with cancer. These include cardiovascular toxicity of certain antineoplastic treatment approaches, [11] a systemic proinflammatory state in patients with cancer that might lead to atrial remodeling, [[12], [13], [14]] and a high perioperative risk of AF in cancer surgery. [14]

Because of the underlying increased risk of both thrombotic events and bleeding in patients with cancer, therapeutic management of AF in this subgroup is especially challenging. [15] Cardiovascular adverse events are crucial drivers of noncancer-related mortality and morbidity, and therefore, the emerging field of cardio-oncology is becoming increasingly relevant today. [16] Prior studies evaluated the risk of AF in patients with cancer and vice versa. [[17], [18], [19], [20], [21], [22], [23]] The majority of those studies were performed on certain cancer types or specific cancer treatments. However, the magnitude of the coprevalence between cancer and AF is still not well defined. Further, differences in coprevalence according to age groups and between specific cancer types are unknown. Therefore, the aim of this nationwide, cross-sectional study was to provide data on coprevalence and relative risk of AF in patients with cancer and to estimate the association with age and cancer type.

## 2 Methods

### 2.1 Study population

The nationwide study population and cohort comprises 8,306,244 Austrian residents. This represents the number of all Austrians below 90 years of age who are covered by health insurance, and accounts for >99.9% of the total Austrian population. [24] Ethical approval and/or informed consent were not required for this nationwide medical claims data.

### 2.2 Data extraction

We have obtained ICD-10 diagnosis codes (main- and next-diagnosis codes) from the Austrian Association of Social Security Providers dataset from the years 2006 to 2007 to explore the prevalence of AF in persons with and without cancer. The ICD-10 codes were introduced in Austria in January 2001.

Patients with AF were defined as having the diagnosis code “I48”—AF and flutter. A history of cancer or active cancer was defined as having at least one ICD-10 diagnosis code starting with the letter “C.” In detail, “C00–C14” —Malignant neoplasms of lip, oral cavity, and pharynx; “C15–C26”—Malignant neoplasms of digestive organs; “C30–C39”—Malignant neoplasms of respiratory and intrathoracic organs; “C40–C41”—Malignant neoplasms of bone and articular cartilage; “C43–C44”—Melanoma and other malignant neoplasms of skin (including basal cell and squamous cell carcinoma); “C45–C49”—Malignant neoplasms of mesothelial and soft tissue; “C50”—Malignant neoplasm of breast; “C51–C58”—Malignant neoplasms of female genital organs; “C60–C63”—Malignant neoplasms of male genital organs; “C64–C68” Malignant neoplasms of urinary tract; “C69–C72”—Malignant neoplasms of eye, brain, and other parts of central nervous system; “C73–C75”—Malignant neoplasms of thyroid and other endocrine glands; “C76–C80”—Malignant neoplasms of ill-defined, secondary, and unspecified sites; “C81–C96” Malignant neoplasms, stated, or presumed to be primary, of lymphoid, hematopoietic and related tissue.

### 2.3 Statistical analysis

The proportion of patients with AF and cancer and vice versa were estimated as point prevalences with 95% binomial exact CIs. This CI gives the estimated range of point prevalences in a population with identical characteristics as our study population. Relative risks of AF in patients with and without cancer were estimated, and summarized across age groups using a random-effects model (Stata routine metan). Subgroup analyses for age categories and tumor types were performed. SPSS 25 (SPSS Inc.) and Stata 15.0 (Stata Corp.) were used to perform all statistical analyses.

## 3 Results

### 3.1 Study cohort

The study cohort consisted of 8,306,244 persons (female: 4,255,119 [51.23%]; male: 4,051,125 [48.77%]). Overall, a cancer diagnosis was present in 158,675 subjects. The corresponding cancer diagnosis code prevalences in the whole study cohort, in females, and in males were 1.91% (95% CI, 1.90-1.92), 1.83% (95% CI, 1.82-1.84), and 2.00% (95% CI, 1.98-2.01), respectively, and increased with age (Supplementary Figure S1). Detailed information regarding cancer prevalences in the whole study cohort, females, and males separated by cancer type is given in Supplementary Table S1. In total, 112,827 patients (59,503 [52.73%] women and 53,324 [47.27%] men) were diagnosed with AF. Accordingly, the prevalence of AF in the whole study cohort, females and males was 1.36% (95% CI, 1.35-1.36), 1.40% (95% CI, 1.39-1.41), and 1.32% (95% CI, 1.31-1.33), respectively, and as well increased with age (Supplementary Figure S2).

### 3.2 Prevalence of atrial fibrillation in persons with versus without a cancer diagnosis code

**Figure 1:**
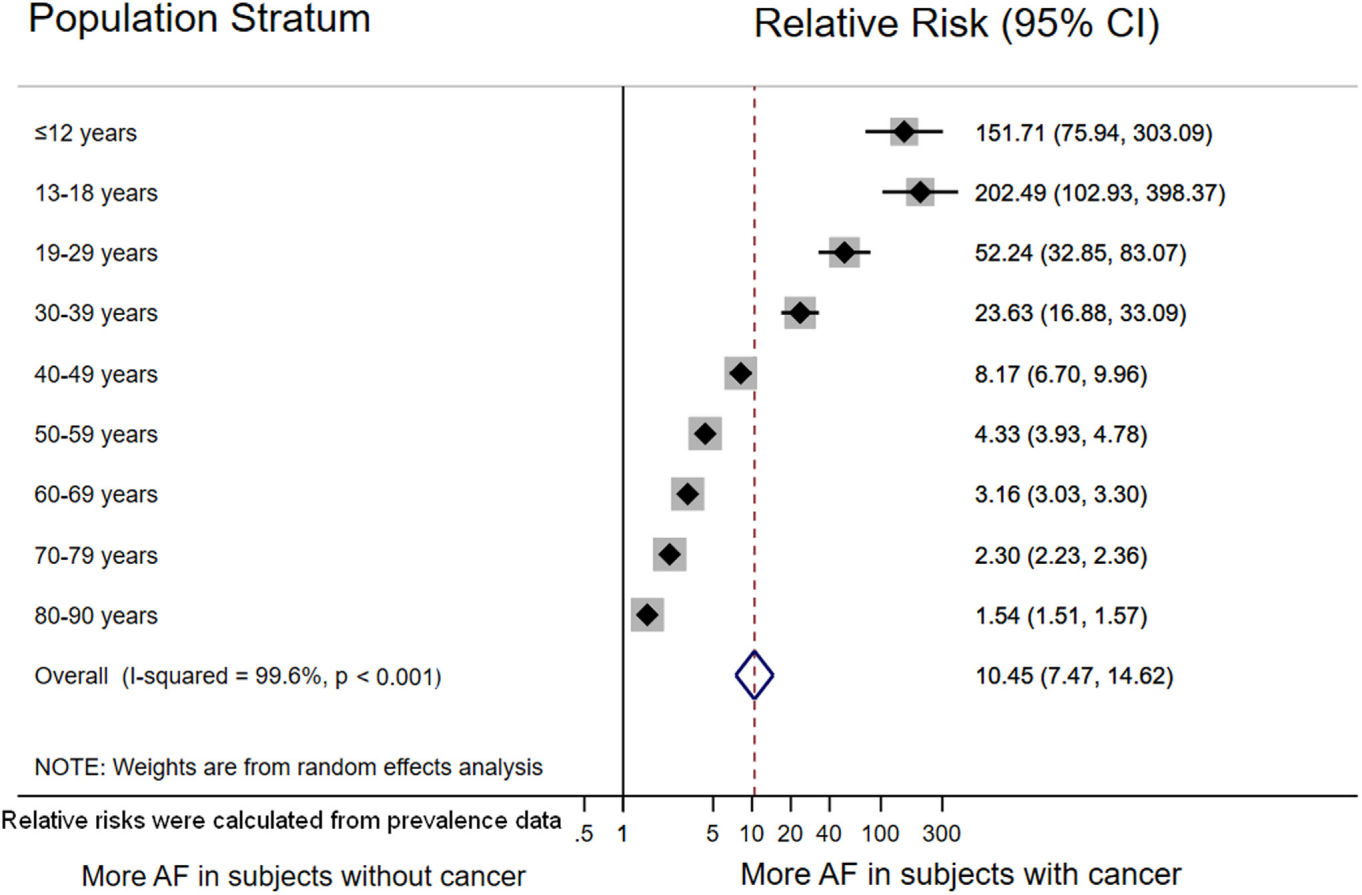
Relative risk of an AF diagnosis code in subjects with and without a cancer diagnosis code. The relative risk of AF declined with increasing age because of the increase in AF prevalence with age in subjects without a cancer diagnosis code. The overall pooled risk ratio was estimated with a random-effects model. Individual age group estimates are depicted as diamonds with 95% CI as bars. Gray boxes surrounding the diamonds are proportional to the weight of the individual age strata within the overall pooled risk ratio. AF, atrial fibrillation

Overall, 15,504 of the 158,675 persons with cancer (point prevalence: 9.77% [95% CI, 9.63-9.92]) and 97,323 of the 8,147,569 persons without cancer (point prevalence: 1.19% [95% CI, 1.19-1.20]) had an AF diagnosis code. Prevalences of an AF diagnosis code in women and men with a cancer diagnosis code were 8.47% and 11.03%, as compared with 1.27% and 1.12% in women and men without a cancer diagnosis code, respectively (Table). This corresponded to a relative risk (RR) of AF and cancer of 8.17 (95% CI, 8.05-8.31; *P* < 0.0001). The age-stratified random-effects relative risk ratio (RE-RRR) of an AF diagnosis code in persons with a cancer diagnosis code was 10.45 (95% CI, 7.47-14.62) (Figure 1). The corresponding RE-RRR for women and men were highly similar (8.64 vs. 8.95), respectively (Supplementary Figure S3).

**Table:** Prevalence of AF diagnosis code in the whole study cohort, all subjects with a cancer diagnosis code, and subjects without cancer diagnosis code separated by age and gender.

	Prevalence of AF in all subjects % (95% CI)	Prevalence of AF in subjects with cancer % (95% CI)	Prevalence of AF in subjects without cancer % (95% CI)
0-90 years	1.36 (1.35-1.36)	9.77 (9.63-9.92)	1.19 (1.19-1.20)
Females	1.40 (1.39-1.40)	8.47 (8.27-8.67)	1.27 (1.26-1.28)
Males	1.32 (1.31-1.33)	11.03 (10.81-11.24)	1.12 (1.10-1.13)
≤12 y	0.01 (0.01-0.01)	1.03 (0.47-1.94)	0.01 (0.01-0.01)
Females	0.01 (0.00-0.01)	1.47 (0.54-3.18)	0.01 (0.00-0.01)
Males	0.01 (0.01-0.01)	0.64 (0.13-1.86)	0.01 (0.01-0.01)
13-18 y	0.01 (0.01-0.01)	1.63 (0.78-2.98)	0.01 (0.01-0.01)
Females	0.01 (0.00-0.01)	1.03 (0.21-2.99)	0.01 (0.00-0.01)
Males	0.01 (0.01-0.01)	2.17 (0.88-4.41)	0.01 (0.01-0.01)
19-29 y	0.03 (0.02-0.03)	1.12 (0.67-1.74)	0.02 (0.02-0.03)
Females	0.02 (0.02-0.02)	0.83 (0.33-1.71)	0.02 (0.02-0.02)
Males	0.03 (0.03-0.04)	1.46 (0.72-2.43)	0.03 (0.02-0.03)
30-39 y	0.05 (0.04-0.05)	1.00 (0.70-1.38)	0.04 (0.04-0.05)
Females	0.03 (0.02-0.03)	0.67 (0.37-1.12)	0.03 (0.02-0.03)
Males	0.06 (0.06-0.07)	1.46 (0.91-2.20)	0.06 (0.05-0.07)
40-49 y	0.12 (0.12-0.13)	0.96 (0.78-1.16)	0.12 (0.11-0.12)
Females	0.07 (0.06-0.07)	0.50 (0.34-0.69)	0.06 (0.06-0.07)
Males	0.18 (0.17-0.19)	1.70 (1.33-2.14)	0.17 (0.16-0.18)
50-59 y	0.49 (0.48-0.51)	2.00 (1.81-2.19)	0.46 (0.45-0.47)
Females	0.27 (0.26-0.28)	1.09 (0.90-1.30)	0.25 (0.24-0.27)
Males	0.72 (0.70-0.074)	2.98 (2.66-3.33)	0.67 (0.65-0.70)
60-69 y	1.86 (1.83-1.89)	5.36 (5.15-5.58)	1.70 (1.67-1.72)
Females	1.31 (1.28-1.34)	3.73 (3.46-4.01)	1.21 (1.18-1.24)
Males	2.47 (2.42-2.51)	6.65 (6.34-6.98)	2.23 (2.19-2.28)
70-79 y	5.88 (5.82-5.94)	12.38 (12.06-12.70)	5.39 (5.33-5.45)
Females	5.03 (4.95-5.10)	10.50 (10.05-10.95)	4.71 (4.64-4.79)
Males	6.98 (6.89-7.08)	13.85 (13.41-14.30)	6.30 (6.21-6.40)
80-90 y	14.11 (14.00-14.22)	20.64 (20.23-21.06)	13.41 (13.29-13.52)
Females	13.31 (13.18-13.45)	19.24 (18.70-19.80)	12.82 (12.68-12.95)
Males	15.84 (15.63-16.05)	22.30 (21.67-22.94)	14.78 (14.57-15.00)

All prevalences are given in percent (%). AF, atrial fibrillation.

### 3.3 Subgroup analysis on risk of atrial fibrillation in patients with versus without cancer by age and cancer type

The RR of AF in patients with versus without cancer strongly decreased with age: Although cancer patients aged 13 to 18 years had the highest RR for AF of 202.49 (95% CI, 102.93-396.37), patients in the 80 to 90 years age category showed a lower RR of 1.54 (95% CI, 1.51-1.57) compared with persons without cancer in the same age group (Figure 1).

**Figure 2:**
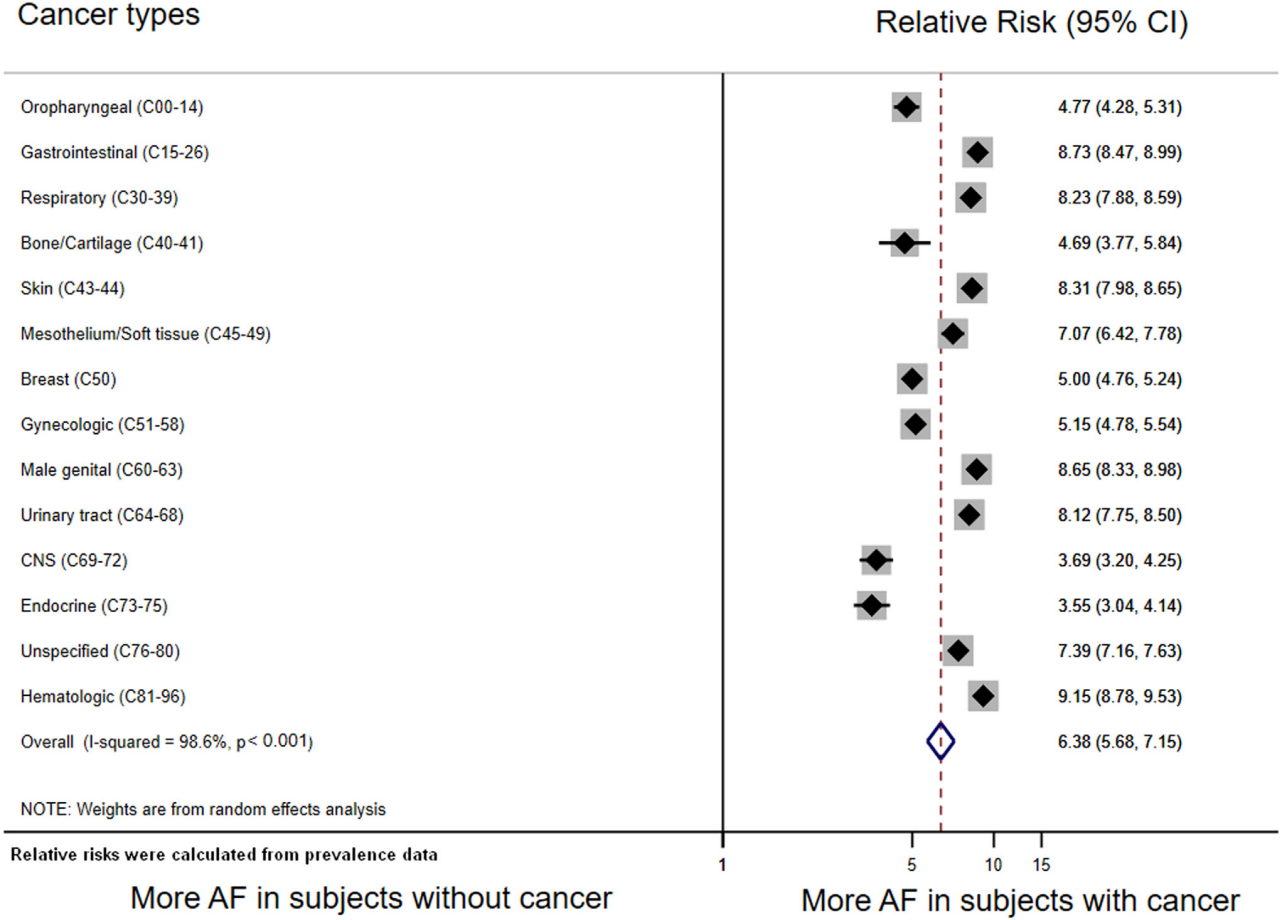
Relative risk of an AF diagnosis code analyzed by cancer type. The overall pooled risk ratio was estimated with a random-effects model. Individual estimates are depicted as diamonds with 95% CI as bars. Gray boxes surrounding the diamonds are proportional to the weight of the individual strata within the overall pooled risk ratio. AF, atrial fibrillation, CNS, central nervous system

Subgroup analysis by cancer type (Figure 2) revealed that RR of AF prevalence was highest in patients with hematologic malignancies (RR = 9.15), followed by patients with gastrointestinal tumors (RR = 8.73), and patients with cancers of the male genital system (RR = 8.65). RR of AF was lowest in patients with endocrine cancer (RR=3.55), tumors of the central nervous system (RR = 3.69), and malignancies of bone and articular cartilage (RR = 4.69).

### 3.4 Prevalence of cancer in patients with an atrial fibrillation diagnosis code

**Figure 3:**
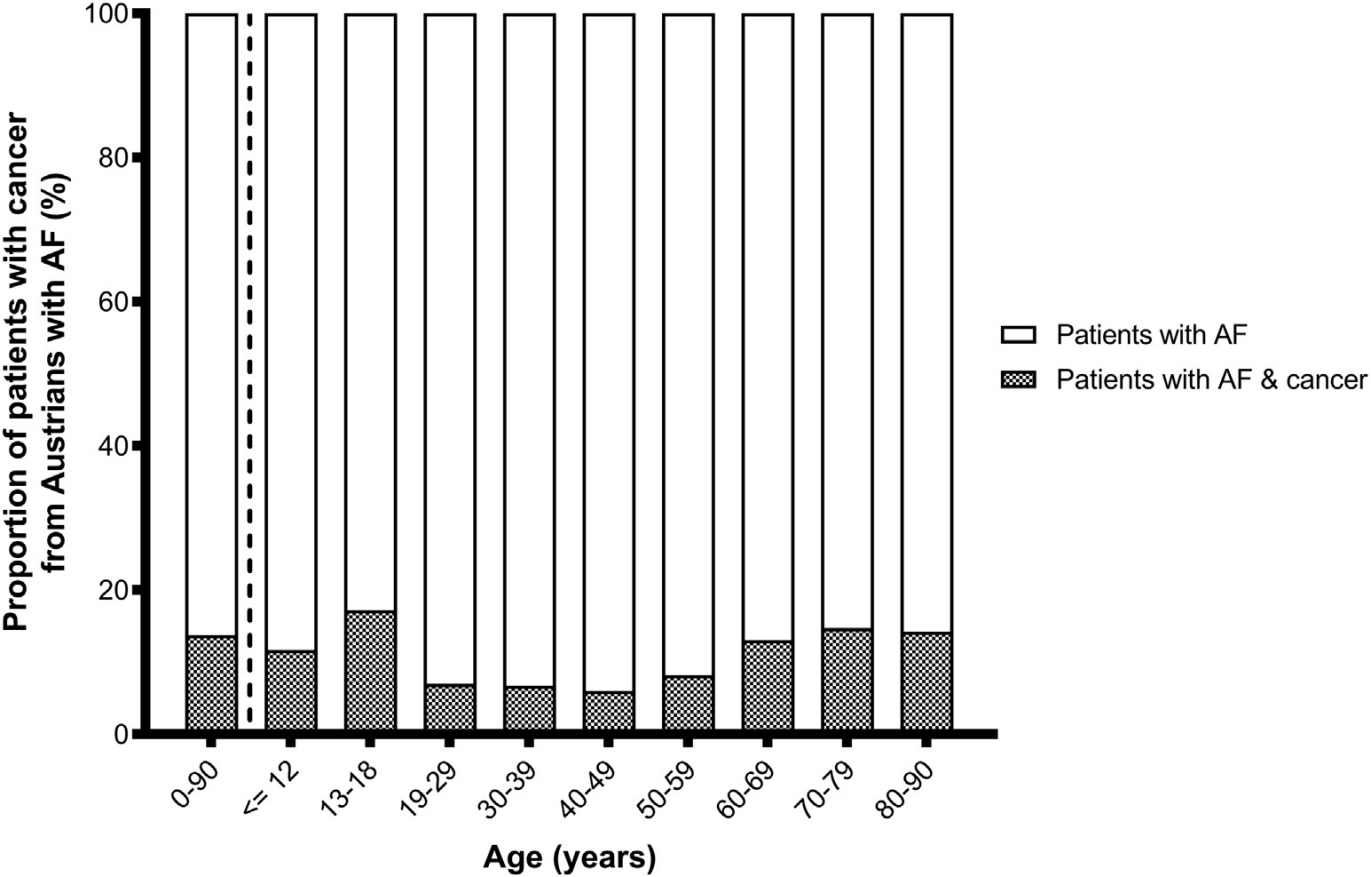
Prevalence of a cancer diagnosis code in persons with a diagnosis code for AF. On the right side of the dashed line results are presented for each age category. AF, atrial fibrillation

In total, 15,504 of the 112,827 (13.74% [95% CI, 13.54-13.94]) patients with an AF diagnosis code also had a documented cancer diagnosis code. The corresponding prevalences for females and males were 11.08% (95% CI, 10.83-11.34) and 16.71% (95% CI, 16.40-17.03), respectively. Detailed information regarding these proportions separated by age and gender is provided in Figure 3 and Supplementary Table S2 and Figure S4. Among patients with AF, gastrointestinal cancer was the most prevalent (3.61%; 95% CI, 3.50-3.72), and bone/cartilage cancer was the least prevalent cancer type (0.07%; 95% CI, 0.05-0.08); Supplementary Table S3.

## 4 Discussion

In this nationwide analysis, we found that AF is highly prevalent in the Austrian cancer population. In detail, around 1 in 10 individuals with cancer had a concurrent AF diagnosis. Random-effects analysis stratified by age group demonstrated that cancer patients were around ten times more likely to be diagnosed with AF than patients without malignancies. Further, the RR of AF was highest in persons with hematologic malignancies and lowest in patients with endocrine malignancies.

Previous studies have already highlighted the interrelation between cancer and AF. [[17], [18], [19], [20], [21],^25^,^26^] Our analysis now provides robust population-level prevalence estimates in patients with cancer and shows that an increased risk of AF exists across different cancer types and age groups. The inverse approach, namely the occurrence of cancer in patients with AF, has similarly been followed by large studies. Melloni et al. [27] found that 1 in 4 patients with AF had a history of cancer, and large cohort studies revealed that risk of cancer diagnosis increases within the first 3 months after new-onset AF. [22,23] Moreover, the occurrence of AF was strongly associated with metastatic cancer. [23] In our epidemiological study, we found a lower but substantial cancer rate of ∼14% in patients with AF.

Taken together, AF and cancer are substantially coprevalent in the population. This might be explained by an increasing prevalence of both diagnoses with higher age [1,2,5,6] and shared risk factors such as comorbidities and lifestyle factors (eg, smoking, alcohol misuse). [9,10] However, the observed higher RR of AF in younger patients with cancer suggests additional underlying mechanisms responsible for the interrelation beyond age and common risk factors. Firstly, cancer is known to induce a systemic proinflammatory state. [28] As of the known role of inflammatory mechanisms in the process of pathological atrial remodeling, cancer might thereby contribute to the development of AF. [29,30] Secondly, certain anticancer treatments, including standard chemotherapy (eg, anthracyclines), targeted therapy (eg, tyrosine kinase inhibitors), or radiotherapy, have known cardiotoxic effects and might increase the risk of developing AF. [[31], [32], [33], [34]] In addition, cancer surgery is a known trigger of perioperative AF. [14,35,36] Patients who underwent lung cancer resection showed high AF rates of up to 20%. [[37], [38], [39]] However, patients with hematologic malignancies, which are treated nonsurgically, presented with the highest RR of AF in our study population. Thus, surgery-related AF might only partly explain the concurrence of cancer and AF, suggesting different underlying risk factors. Thirdly, alterations of the autonomic nervous systems because of increased sympathetic activity caused by pain or emotional distress have been described as a possible mechanism for AF. [40] However, future research is needed to better understand the interplay between these mechanisms and explore the factors responsible for the high prevalence of AF in patients with cancer.

Patient management of AF and an underlying cancer diagnosis is challenging because of an increased risk of stroke and major bleeding. Further, AF diagnosis has a detrimental effect on the prognosis of patients with cancer. [19,41] Unfortunately, current clinical prediction tools, eg, CHA_2_DS_2_-VASc and HAS-BLED score, do not consider cancer diagnosis and may not be suitable for patients with cancer. Nevertheless, in the absence of better tools, the CHA_2_DS_2_-VASc score is recommended. [42] Moreover, data from a nationwide Swedish study could show net cerebrovascular benefit of oral anticoagulation with regard to stroke and bleeding in patients with AF and active cancer, demonstrating the importance of antithrombotic therapy strategies in patients with cancer. [43] Therefore, specific guidance on antithrombotic therapy for stroke prevention in patients with cancer is needed to improve patient care in this challenging and emerging patient population. [44,45]

Our study has several limitations that need to be acknowledged. First, the temporal relationship between cancer and AF could not be investigated based on the available prevalence data. Secondly, inaccuracies in ICD-10 diagnosis coding may introduce error into our prevalence estimates. However, population-level data on both, AF and cancer, show similar rates compared with other studies. [46,47] Thirdly, detection bias might further influence our results. As patients with cancer frequently attend medical visits, detection of asymptomatic AF is more likely in patients with cancer than in those without cancer. Fourthly, given the age of our dataset, prevalence and management of both cancer and AF might have changed since data collection. Additionally, our study is limited by single-country data without available information on race and socioeconomic status.

In conclusion, we demonstrate that AF is a frequent codiagnosis in patients with cancer, and risk of AF is substantially higher in patients with cancer across all age groups and tumor types. This growing patient population provides new challenges to the field of cardio-oncology. Therefore, more research is needed to guide treatment and management of AF in patients with cancer and to understand the underlying causality for the coprevalence of cancer and AF.
