# Supplementary material for: Atrial fibrillation and cancer: prevalence and relative risk from a nationwide study
Source: Res Pract Thromb Haemost. 2022 Dec 23;7(1):100026. doi: 10.1016/j.rpth.2022.100026 (PMC9986100; doi:10.1016/j.rpth.2022.100026)

**Atrial fibrillation and cancer: prevalence and relative risk from a nationwide study – supplemental material**

Running title: Atrial fibrillation and cancer: prevalence and relative risk

**Cihan Ay^a^**, Ella Grilz^a^, Stephan Nopp^a^, Florian Moik^a,b^, Oliver Königsbrügge^a^, Peter Klimek^c,d^, Stefan Thurner^c,d,e^, Florian Posch^b^, Ingrid Pabinger^a^

*^a^ Department of Medicine I, Medical University of Vienna, Vienna, Austria*

*^b^ Department of Internal Medicine, Medical University of Graz, Graz, Austria*

*^c^ Section for Science of Complex Systems, CEMSIIS, Medical University of Vienna, Vienna, Austria*

*^d^ Complexity Science Hub Vienna, Vienna, Austria*

*^e^ Santa Fe Institute, Santa Fe, NM, USA*

Address for correspondence: Univ.-Prof. Priv.-Doz. Dr. Cihan Ay

Clinical Division of Hematology and Hemostaseology

Department of Medicine I, Medical University of Vienna

Waehringer Guertel 18-20, A-1090 Vienna, Austria

Phone number: +43 1 40400 44100

Fax number: +43 1 40400 403000

Email: cihan.ay@meduniwien.ac.at

# Supplementary Table and Figures

## Supplementary Table S1.

**Prevalences of a cancer diagnosis code in persons separated by cancer type and age group**. ICD-10 diagnosis codes used are presented for each cancer type.

| **Cancer type** | **Prevalence of Cancer % (95% Confidence Intervals)** | | | | | | | | | |
| --- | --- | --- | --- | --- | --- | --- | --- | --- | --- | --- |
|  | **0-90 years** | **≤12 years** | **13-18 years** | **19-29 years** | **30-39 years** | **40-49 years** | **50-59 years** | **60-69 years** | **70-79 years** | **80-90 years** |
| **All types** | **1.91**  **(1.90-1.92)** | **0.09**  **(0.08-0.09)** | **0.09**  **(0.08-0.09)** | **0.16**  **(0.15-0.17)** | **0.30**  **(0.29-0.31)** | **0.78**  **(0.76-0.79)** | **2.02**  **(1.99-2.04)** | **4.52**  **(4.48-4.56)** | **7.01**  **(6.94-7.07)** | **9.72**  **(9.63-9.82)** |
| **Female** | **1.83**  **(1.82-1.84)** | **0.08**  **(0.08-0.09)** | **0.09**  **(0.08-0.10)** | **0.16**  **(0.15-0.17)** | **0.35**  **(0.34-0.37)** | **0.97**  **(0.95-0.99)** | **2.08**  **(2.04-2.11)** | **3.82**  **(3.77-3.87)** | **5.45**  **(5.37-5.52)** | **7.72**  **(7.61-7.82)** |
| **Male** | **2.00**  **(1.98-2.01)** | **0.09**  **(0.08-0.10)** | **0.9**  **(0.8-0.10)** | **0.16**  **(0.15-0.17)** | **0.26**  **(0.24-0.27)** | **0.59**  **(0.57-0.60)** | **1.96**  **(1.92-1.99)** | **5.29**  **(5.22-5.35)** | **9.02**  **(8.91-9.13)** | **14.06**  **(13.87-14.26)** |
| Oropharyngeal  (C00-14) | 0.06  (0.06-0.06) | 0.00  (0.00-0.00) | 0.00  (0.00-0.00) | 0.00  (0.00-0.01) | 0.01  (0.00-0.01) | 0.03  (0.03-0.03) | 0.11  (0.11-0.12) | 0.17  (0.17-0.18) | 0.14  (0.13-0.15) | 0.14  (0.12-0.15) |
| Female | 0.03  (0.03-0.03) | 0.00  (0.00-0.00) | 0.00  (0.00-0.00) | 0.00  (0.00-0.00) | 0.00  (0.00-0.01) | 0.02  (0.01-0.02) | 0.05  (0.05-0.06) | 0.08  (0.07-0.09) | 0.07  (0.07-0.08) | 0.10  (0.09-0.11) |
| Male | 0.08  (0.08-0.09) | 0.00  (0.00-0.00) | 0.00  (0.00-0.00) | 0.00  (0.00-0.01) | 0.01  (0.00-0.01) | 0.04  (0.04-0.05) | 0.18  (0.16-0.19) | 0.28  (0.26-0.29) | 0.23  (0.22-0.25) | 0.21  (0.19-0.24) |
| Gastrointestinal  (C15-29) | 0.43  (0.42-0.43) | 0.00  (0.00-0.01) | 0.01  (0.00-0.01) | 0.01  (0.01-0.01) | 0.03  (0.03-0.03) | 0.10  (0.10-0.11) | 0.36  (0.35-0.38) | 1.03  (1.01-1.05) | 1.81  (1.78-1.85) | 2.51  (2.46-2.56) |
| Female | 0.37  (0.36-0.37) | 0.00  (0.00-0.01) | 0.00  (0.00-0.01) | 0.01  (0.01-0.01) | 0.03  (0.03-0.04) | 0.09  (0.09-0.10) | 0.29  (0.27-0.30) | 0.72  (0.70-0.74) | 1.30  (1.27-1.34) | 2.05  (1.99-2.10) |
| Male | 0.49  (0.49-0.50) | 0.00  (0.00-0.01) | 0.01  (0.00-0.01) | 0.01  (0.01-0.02) | 0.03  (0.03-0.04) | 0.11  (0.10-0.12) | 0.45  (0.43-0.46) | 1.36  (1.33-1.4) | 2.47  (2.41-2.53) | 3.51  (3.41-3.62) |
| Respiratory  (C30-39) | 0.20  (0.20-0.21) | 0.00  (0.00-0.00) | 0.00  (0.00-0.00) | 0.01  (0.01-0.01) | 0.01  (0.01-0.01) | 0.06  (0.06-0.06) | 0.28  (0.27-0.29) | 0.61  (0.59-0.63) | 0.75  (0.72-0.77) | 0.72  (0.69-0.75) |
| Female | 0.14  (0.13-0.14) | 0.00  (0.00-0.00) | 0.00  (0.00-0.00) | 0.01  (0.00-0.01) | 0.01  (0.01-0.01) | 0.05  (0.04-0.06) | 0.20  (0.18-0.21) | 0.37  (0.35-0.39) | 0.40  (0.38-0.42) | 0.44  (0.41-0.47) |
| Male | 0.27  (0.27-0.28) | 0.00  (0.00-0.01) | 0.00  (0.00-0.01) | 0.01  (0.01-0.01) | 0.02  (0.01-0.02) | 0.07  (0.07-0.08) | 0.36  (0.34-0.38) | 0.87  (0.85-0.90) | 1.19  (1.15-1.24) | 1.33  (1.26-1.4) |
| Bone/Cartilage  (C40-41) | 0.01  (0.01-0.02) | 0.00  (0.00-0.01) | 0.01  (0.01-0.01) | 0.01  (0.01-0.01) | 0.01  (0.00-0.01) | 0.01  (0.01-0.01) | 0.02  (0.01-0.02) | 0.03  (0.02-0.03) | 0.04  (0.03-0.04) | 0.04  (0.03-0.05) |
| Female | 0.01  (0.01-0.01) | 0.00  (0.00-0.01) | 0.01  (0.00-0.01) | 0.01  (0.01-0.01) | 0.01  (0.00-0.01) | 0.01  (0.01-0.01) | 0.01  (0.01-0.02) | 0.02  (0.02-0.03) | 0.03  (0.02-0.04) | 0.03  (0.03-0.04) |
| Male | 0.02  (0.01-0.02) | 0.00  (0.00-0.01) | 0.01  (0.01-0.01) | 0.01  (0.01-0.01) | 0.00  (0.00-0.01) | 0.01  (0.01-0.01) | 0.02  (0.02-0.03) | 0.03  (0.03-0.04) | 0.05  (0.04-0.06) | 0.05  (0.04-0.07) |
| Skin  (C43-44) | 0.23  (0.23-0.24) | 0.00  (0.00-0.00) | 0.01  (0.00-0.01) | 0.02  (0.01-0.02) | 0.04  (0.03-0.04) | 0.09  (0.08-0.09) | 0.18  (0.17-0.19) | 0.43  (0.42-0.44) | 0.86  (0.84-0.88) | 1.75  (1.71-1.79) |
| Female | 0.23  (0.22-0.23) | 0.00  (0.00-0.01) | 0.01  (0.00-0.01) | 0.02  (0.02-0.02) | 0.04  (0.04-0.05) | 0.10  (0.09-0.10) | 0.17  (0.16-0.18) | 0.36  (0.34-0.37) | 0.69  (0.66-0.72) | 1.46  (1.41-1.50) |
| Male | 0.24  (0.23-0.24) | 0.00  (0.00-0.00) | 0.00  (0.00-0.01) | 0.01  (0.01-0.01) | 0.03  (0.03-0.04) | 0.08  (0.07-0.08) | 0.18  (0.17-0.19) | 0.51  (0.49-0.53) | 1.08  (1.04-1.12) | 2.38  (2.30-2.47) |
| Mesothelium/  Soft tissue  (C45-49) | 0.05  (0.05-0.05) | 0.01  (0.01-0.01) | 0.01  (0.00-0.01) | 0.01  (0.01-0.01) | 0.01  (0.01-0.01) | 0.02  (0.02-0.02) | 0.05  (0.04-0.05) | 0.11  (0.10-0.11) | 0.17  (0.16-0.18) | 0.2  (0.18-0.21) |
| Female | 0.05  (0.05-0.05) | 0.01  (0.00-0.01) | 0.00  (0.00-0.01) | 0.01  (0.01-0.01) | 0.01  (0.01-0.01) | 0.02  (0.02-0.03) | 0.06  (0.05-0.06) | 0.11  (0.10-0.12) | 0.17  (0.16-0.19) | 0.19  (0.17-0.21) |
| Male | 0.04  (0.04-0.04) | 0.01  (0.01-0.01) | 0.01  (0.00-0.01) | 0.01  (0.00-0.01) | 0.01  (0.01-0.01) | 0.02  (0.02-0.02) | 0.04  (0.04-0.05) | 0.10  (0.09-0.11) | 0.17  (0.16-0.19) | 0.21  (0.19-0.24) |
| Breast  (C50) | 0.28  (0.28-0.28) | 0.00  (0.00-0.00) | 0.00  (0.00-0.00) | 0.01  (0.01-0.01) | 0.05  (0.05-0.06) | 0.21  (0.20-0.22) | 0.41  (0.40-0.43) | 0.69  (0.67-0.71) | 0.79  (0.77-0.81) | 1.07  (1.04-1.11) |
| Female | 0.54  (0.53-0.54) | 0.00  (0.00-0.01) | 0.00  (0.00-0.01) | 0.02  (0.01-0.02) | 0.11  (0.10-0.12) | 0.42  (0.41-0.44) | 0.81  (0.78-0.83) | 1.30  (1.27-1.34) | 1.37  (1.33-1.41) | 1.55  (1.50-1.60) |
| Male | 0.01  (0.01-0.01) | 0.00  (0.00-0.00) | 0.00  (0.00-0.00) | 0.00  (0.00-0.00) | 0.00  (0.00-0.00) | 0.00  (0.00-0.00) | 0.01  (0.01-0.01) | 0.02  (0.02-0.03) | 0.03  (0.03-0.04) | 0.05  (0.03-0.06) |
| Gynecologic  (C51-58) | 0.11  (0.11-0.12) | 0.00  (0.00-0.00) | 0.00  (0.00-0.00) | 0.01  (0.01-0.01) | 0.03  (0.02-0.03) | 0.06  (0.06-0.07) | 0.15  (0.14-0.16) | 0.26  (0.25-0.27) | 0.38  (0.36-0.39) | 0.49  (0.47-0.51) |
| Female | 0.22  (0.22-0.23) | 0.00  (0.00-0.01) | 0.01  (0.00-0.01) | 0.02  (0.01-0.02) | 0.05  (0.05-0.06) | 0.13  (0.12-0.14) | 0.30  (0.28-0.31) | 0.50  (0.48-0.52) | 0.66  (0.64-0.69) | 0.71  (0.68-0.74) |
| Male | 0.00  (0.00-0.00) | 0.00  (0.00-0.00) | 0.00  (0.00-0.00) | 0.00  (0.00-0.00) | 0.00  (0.00-0.00) | 0.00  (0.00-0.00) | 0.00  (0.00-0.00) | 0.00  (0.00-0.00) | 0.00  (0.00-0.01) | 0.00  (0.00-0.01) |
| Male genital  (C60-63) | 0.26  (0.25-0.26) | 0.00  (0.00-0.01) | 0.00  (0.00-0.01) | 0.02  (0.02-0.02) | 0.04  (0.04-0.04) | 0.05  (0.05-0.06) | 0.18  (0.18-0.19) | 0.67  (0.65-0.68) | 1.13  (1.10-1.15) | 1.32  (1.29-1.36) |
| Female | 0.01  (0.01-0.01) | 0.00  (0.00-0.00) | 0.00  (0.00-0.00) | 0.00  (0.00-0.00) | 0.00  (0.00-0.00) | 0.00  (0.00-0.00) | 0.00  (0.00-0.00) | 0.01  (0.01-0.02) | 0.05  (0.04-0.06) | 0.07  (0.06-0.08) |
| Male | 0.51  (0.51-0.52) | 0.01  (0.00-0.01) | 0.01  (0.00-0.01) | 0.04  (0.04-0.05) | 0.08  (0.07-0.08) | 0.10  (0.09-0.11) | 0.37  (0.36-0.39) | 1.38  (1.35-1.41) | 2.52  (2.46-2.58) | 4.05  (3.94-4.16) |
| Urinary tract  (C64-68) | 0.18  (0.18-0.18) | 0.01  (0.00-0.01) | 0.00  (0.00-0.00) | 0.00  (0.00-0.01) | 0.01  (0.01-0.01) | 0.04  (0.04-0.05) | 0.14  (0.13-0.15) | 0.40  (0.39-0.41) | 0.81  (0.78-0.83) | 1.09  (1.06-1.12) |
| Female | 0.11  (0.11-0.11) | 0.01  (0.00-0.01) | 0.00  (0.00-0.00) | 0.01  (0.00-0.01) | 0.01  (0.00-0.01) | 0.03  (0.03-0.04) | 0.08  (0.07-0.09) | 0.21  (0.20-0.22) | 0.44  (0.42-0.46) | 0.57  (0.54-0.60) |
| Male | 0.25  (0.25-0.26) | 0.01  (0.00-0.01) | 0.00  (0.00-0.01) | 0.00  (0.00-0.01) | 0.01  (0.01-0.02) | 0.06  (0.05-0.06) | 0.20  (0.19-0.21) | 0.61  (0.59-0.63) | 1.28  (1.23-1.32) | 2.22  (2.14-2.31) |
| Central nervous system  (C69-72) | 0.04  (0.04-0.05) | 0.02  (0.02-0.02) | 0.02  (0.02-0.03) | 0.02  (0.01-0.02) | 0.02  (0.02-0.02) | 0.03  (0.03-0.03) | 0.05  (0.05-0.06) | 0.09  (0.08-0.10) | 0.11  (0.10-0.12) | 0.11  (0.10-0.12) |
| Female | 0.04  (0.04-0.04) | 0.02  (0.02-0.03) | 0.03  (0.02-0.03) | 0.01  (0.01-0.02) | 0.02  (0.01-0.02) | 0.03  (0.03-0.03) | 0.05  (0.04-0.06) | 0.08  (0.07-0.09) | 0.09  (0.08-0.10) | 0.09  (0.08-0.10) |
| Male | 0.05  (0.04-0.05) | 0.02  (0.02-0.03) | 0.02  (0.02-0.03) | 0.02  (0.01-0.02) | 0.02  (0.01-0.02) | 0.03  (0.03-0.04) | 0.06  (0.05-0.07) | 0.10  (0.09-0.11) | 0.13  (0.12-0.15) | 0.14  (0.12-0.16) |
| Endocrine  (C73-75) | 0.04  (0.04-0.04) | 0.01  (0.01-0.01) | 0.00  (0.00-0.00) | 0.01  (0.01-0.02) | 0.02  (0.02-0.03) | 0.04  (0.04-0.04) | 0.06  (0.06-0.07) | 0.08  (0.08-0.09) | 0.08  (0.07-0.08) | 0.07  (0.06-0.08) |
| Female | 0.05  (0.05-0.05) | 0.01  (0.00-0.01) | 0.00  (0.00-0.01) | 0.02  (0.02-0.03) | 0.03  (0.03-0.04) | 0.06  (0.05-0.06) | 0.08  (0.07-0.09) | 0.10  (0.09-0.11) | 0.09  (0.08-0.10) | 0.08  (0.07-0.09) |
| Male | 0.03  (0.02-0.03) | 0.01  (0.01-0.01) | 0.00  (0.00-0.00) | 0.01  (0.01-0.01) | 0.01  (0.01-0.02) | 0.02  (0.02-0.02) | 0.04  (0.04-0.05) | 0.07  (0.06-0.07) | 0.06  (0.05-0.07) | 0.07  (0.05-0.08) |
| Unspecified  (C76-80) | 0.44  (0.44-0.45) | 0.01  (0.01-0.01) | 0.01  (0.01-0.01) | 0.02  (0.02-0.03) | 0.05  (0.05-0.06) | 0.17  (0.16-0.17) | 0.50  (0.49-0.51) | 1.12  (1.10-1.14) | 1.69  (1.66-1.72) | 2.12  (2.07-2.16) |
| Female | 0.44  (0.43-0.44) | 0.01  (0.01-0.01) | 0.01  (0.01-0.01) | 0.02  (0.02-0.02) | 0.06  (0.05-0.06) | 0.20  (0.19-0.21) | 0.51  (0.49-0.53) | 1.00  (0.98-1.03) | 1.39  (1.35-1.43) | 1.77  (1.72-1.83) |
| Male | 0.45  (0.44-0.46) | 0.01  (0.01-0.01) | 0.01  (0.00-0.01) | 0.03  (0.02-0.03) | 0.05  (0.04-0.05) | 0.13  (0.13-0.14) | 0.49  (0.47-0.51) | 1.24  (1.21-1.27) | 2.08  (2.03-2.14) | 2.86  (2.76-2.95) |
| Haematologic  (C81-96) | 0.20  (0.19-0.20) | 0.03  (0.02-0.03) | 0.03  (0.03-0.03) | 0.04  (0.04-0.05) | 0.04  (0.04-0.05) | 0.08  (0.07-0.08) | 0.18  (0.17-0.19) | 0.4  (0.39-0.42) | 0.73  (0.71-0.75) | 1.03  (1.00-1.07) |
| Female | 0.19  (0.18-0.19) | 0.03  (0.02-0.03) | 0.02  (0.02-0.03) | 0.04  (0.03-0.04) | 0.04  (0.04-0.05) | 0.07  (0.06-0.08) | 0.15  (0.14-0.16) | 0.34  (0.32-0.36) | 0.61  (0.58-0.63) | 0.89  (0.85-0.92) |
| Male | 0.21  (0.21-0.21) | 0.03  (0.02-0.03) | 0.03  (0.03-0.04) | 0.05  (0.04-0.05) | 0.05  (0.04-0.05) | 0.09  (0.08-0.10) | 0.21  (0.19-0.22) | 0.48  (0.46-0.50) | 0.89  (0.85-0.92) | 1.35  (1.29-1.42) |

## Supplementary Table S2.

Prevalence of cancer in all Austrians **with an AF diagnosis code, separated by age and gender.** All results are given in percentages.

AF = Atrial fibrillation; CI = Confidence interval

| **Age group** | **Prevalence of cancer diagnosis code in subjects with an AF diagnosis code % (95% binomial exact CI)** |
| --- | --- |
| **0-90 years**  **Female**  **Male** | **13.74 (13.54-13.94)**  **11.08 (10.83-11.34)**  **16.71 (16.40-17.03)** |
| ≤12 years  Female  Male | 11.69 (5.49-21.03)  17.14 (6.56-33.65)  7.14 (1.50-19.48) |
| 13-18 years  Female  Male | 17.24 (8.59-29.43)  14.29 (3.05-36.43)  18.92 (7.96-35.16) |
| 19-29 years  Female  Male | 7.01 (4.27-10.73)  6.48 (2.65-12.90)  7.36 (3.86-12.51) |
| 30-39 years  Female  Male | 6.72 (4.75-9.18)  8.59 (4.78-13.99)  5.90 (3.73-8.79) |
| 40-49 years  Female  Male | 6.00 (4.93-7.23)  7.32 (5.09-10.12)  5.53 (4.34-6.94) |
| 50-59 years  Female  Male | 8.19 (7.46-8.96)  8.39 (7.01-9.94)  8.11 (7.26-9.03) |
| 60-69 years  Female  Male | 13.03 (12.53-13.54)  10.90 (10.15-11.70)  14.26 (13.61-14.93) |
| 70-79 years  Female  Male | 14.75 (14.37-15.12)  11.37 (10.89-11.86)  17.89 (17.33-18.46) |
| 80-90 years  Female  Male | 14.22 (13.92-14.52)  11.15 (10.82-11.49)  19.80 (19.23-20.37) |

## Supplementary Table S3.

Prevalence of cancer in all Austrians **with an AF diagnosis code, separated by cancer type.**

AF = Atrial fibrillation; CI = Confidence interval

| **Cancer type** | **Proportion of cancer diagnosis code in subjects with an AF diagnosis code (95% CI)** |
| --- | --- |
| **Overall**  **Female**  **Male** | **13.74 (13.54-13.94)**  **11.08 (10.83-11.34)**  **16.71 (16.40-17.03)** |
| Oropharyngeal (C00-14)  Female  Male | 0.27 (0.24-0.30)  0.13 (0.10-0.16)  0.43 (0.38-0.49) |
| Gastrointestinal (C15-26)  Female  Male | 3.61 (3.50-3.72)  2.78 (2.65-2.91)  4.54 (4.36-4.72) |
| Respiratory (C30-39)  Female  Male | 1.64 (1.57-1.72)  0.89 (0.82-0.97)  2.48 (2.35-2.61) |
| Bone/Cartilage (C40-41)  Female  Male | 0.07 (0.05-0.08)  0.06 (0.04-0.08)  0.08 (0.05-0.10) |
| Skin (C43-44)  Female  Male | 1.90 (1.83-1.97)  1.70 (1.60-1.80)  2.14 (2.01-2.26) |
| Mesothelium/Soft tissue (C45-49)  Female  Male | 0.33 (0.30-0.37)  0.33 (0.29-0.38)  0.33 (0.28-0.38) |
| Breast (C50)  Female  Male | 1.38 (1.31-1.45)  2.53 (2.40-2.66)  0.10 (0.07-0.13) |
| Gynecologic (C51-58)  Female  Male | 0.58 (0.54-0.63)  1.09 (1.01-1.18)  0.01 (0.00-0.02) |
| Male genital (C60-63)  Female  Male | 2.17 (2.08-2.25)  0.09 (0.06-0.11)  4.49 (4.32-4.67) |
| Urinary tract (C64-68)  Female  Male | 1.44 (1.37-1.51)  0.80 (0.73-0.87)  2.15 (2.03-2.28) |
| CNS (C69-72)  Female  Male | 0.16 (0.14-0.19)  0.15 (0.12-0.18)  0.17 (0.14-0.21) |
| Endocrine (C73-75)  Female  Male | 0.14 (0.12-0.16)  0.17 (0.14-0.21)  0.11 (0.08-0.14) |
| Unspecified (C76-80)  Female  Male | 3.19 (3.09-3.30)  2.60 (2.48-2.74)  3.85 (3.69-4.02) |
| Hematologic (C81-96)  Female  Male | 1.77 (1.70-1.85)  1.52 (1.42-1.62)  2.06 (1.94-2.19) |

## Supplementary Figure S1. Prevalence of a cancer diagnosis code in the Austrian population stratified by age.

Cancer prevalence increased with age from 0.09% in subjects aged ≤12 years to 9.72% in subjects aged 80-90 years.

Note the scaling of x-axis from 0% to 15% of cancer prevalence.

**
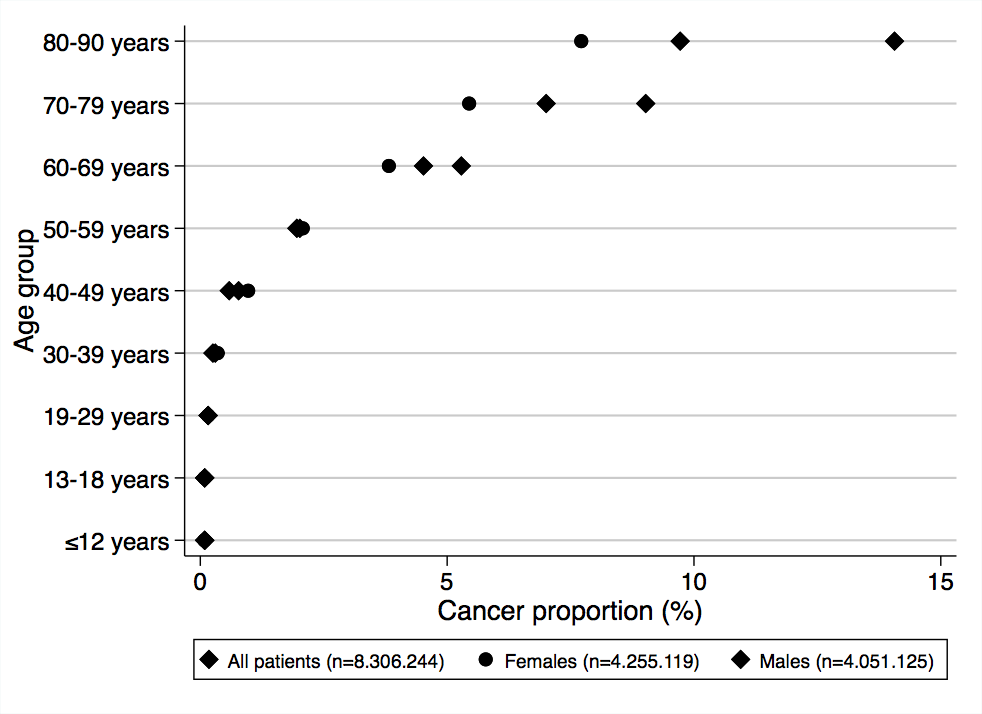
**

## Supplementary Figure S2. Prevalence of an AF diagnosis code in the Austrian population stratified by age.

Note the scaling of x-axis from 0% to 15% of AF prevalence.

AF = Atrial fibrillation

**
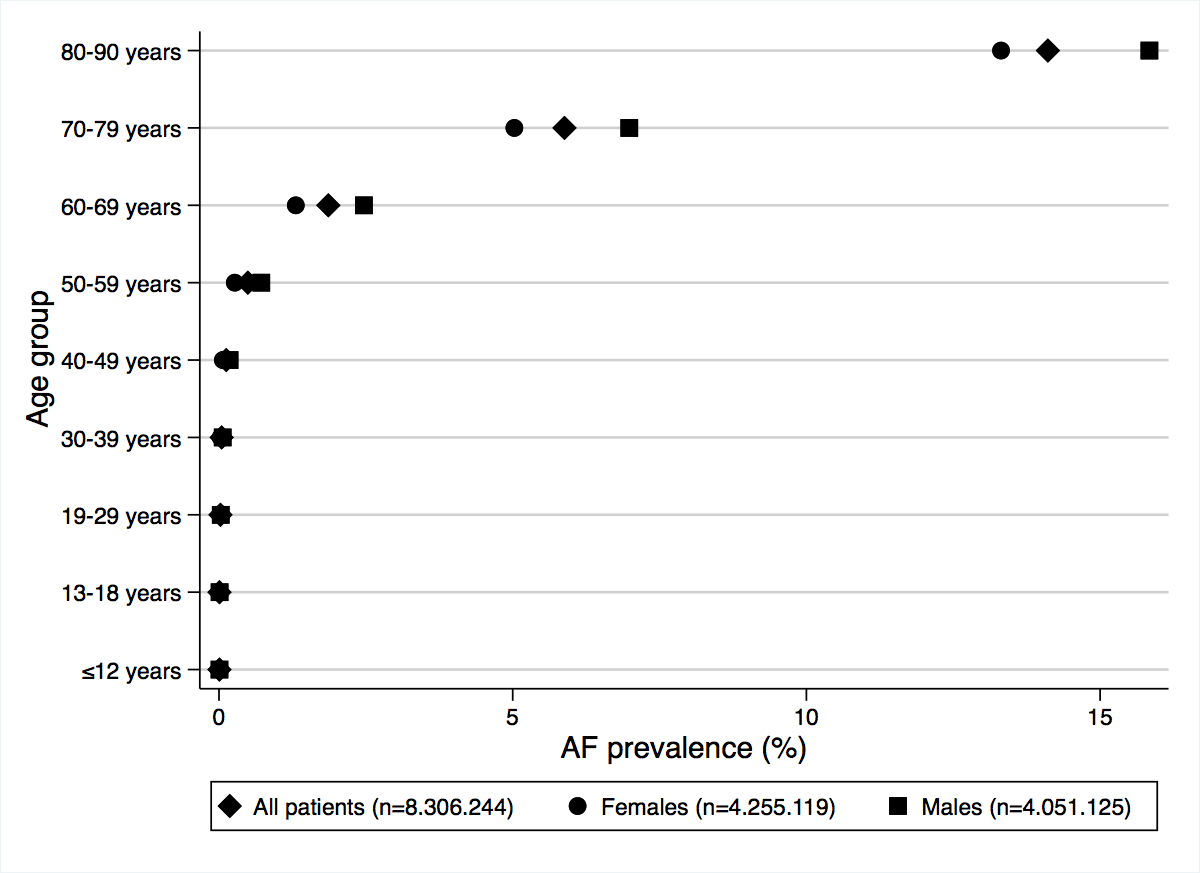
**

## Supplementary Figure S3. Relative risk of an AF diagnosis code in subjects with and without a cancer diagnosis code for females (a) and males (b).

The relative risk of AF declined with increasing age due to the increase in AF prevalence with age in subjects without a cancer diagnosis code. The overall pooled risk ratio was estimated with a random-effects model. Individual age group estimates are depicted as diamonds with 95% confidence intervals as bars. Grey boxes surrounding the diamonds are proportional to the weight of the individual age strata within the overall pooled risk ratio. Abbreviations: AF = Atrial fibrillation, CI = 95% confidence interval

**a)**
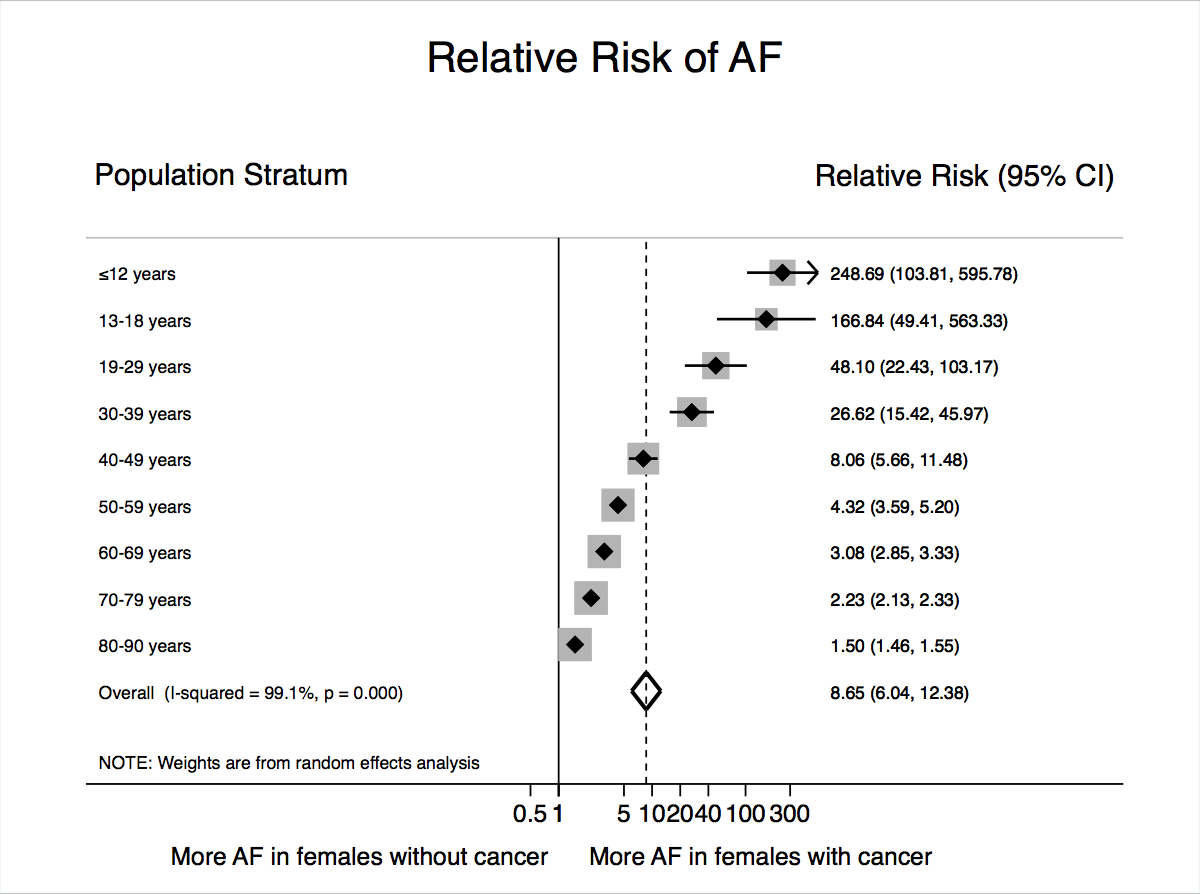


**b)**
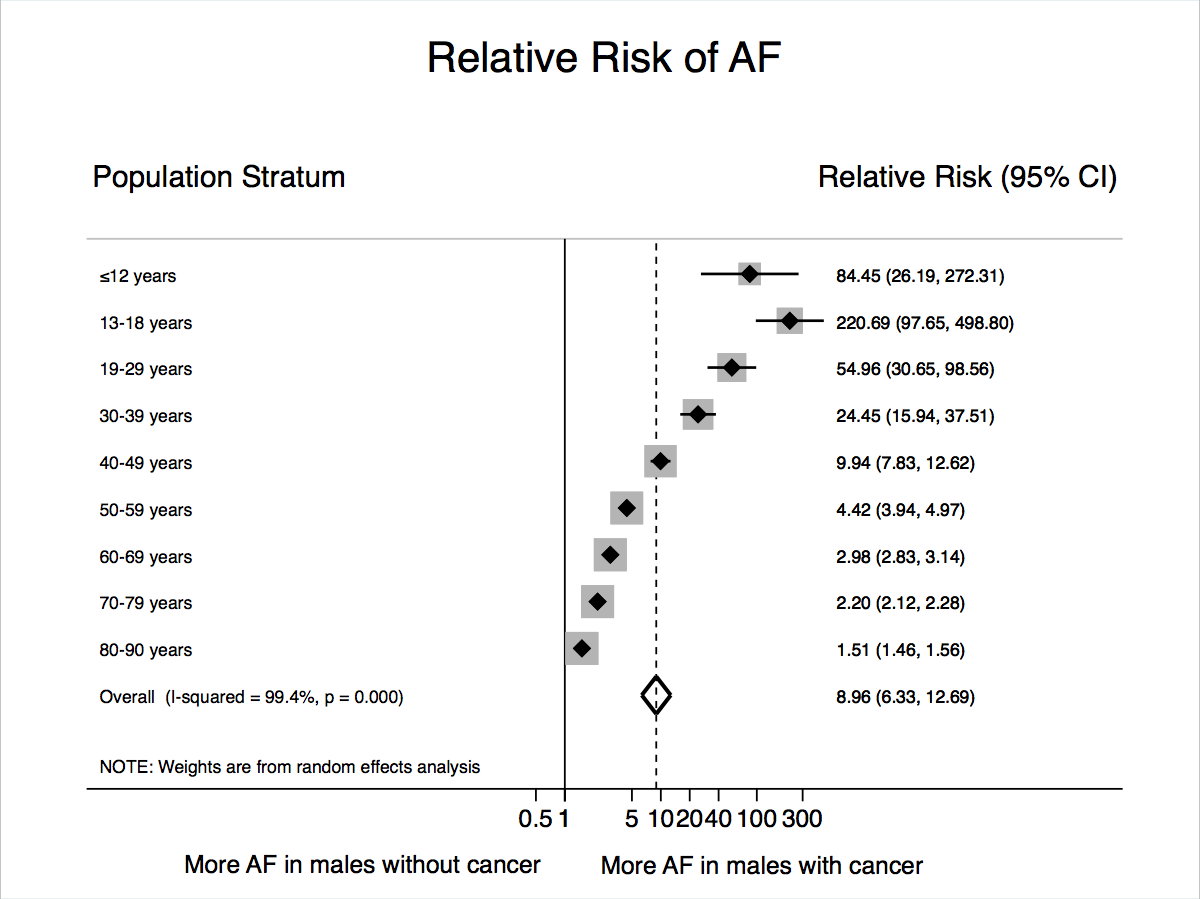


## Supplementary Figure S4. Prevalence of a cancer diagnosis code in persons with a diagnosis code for AF stratified by age and sex.

On the right side of the dashed line results are presented for each age category.

AF = Atrial fibrillation


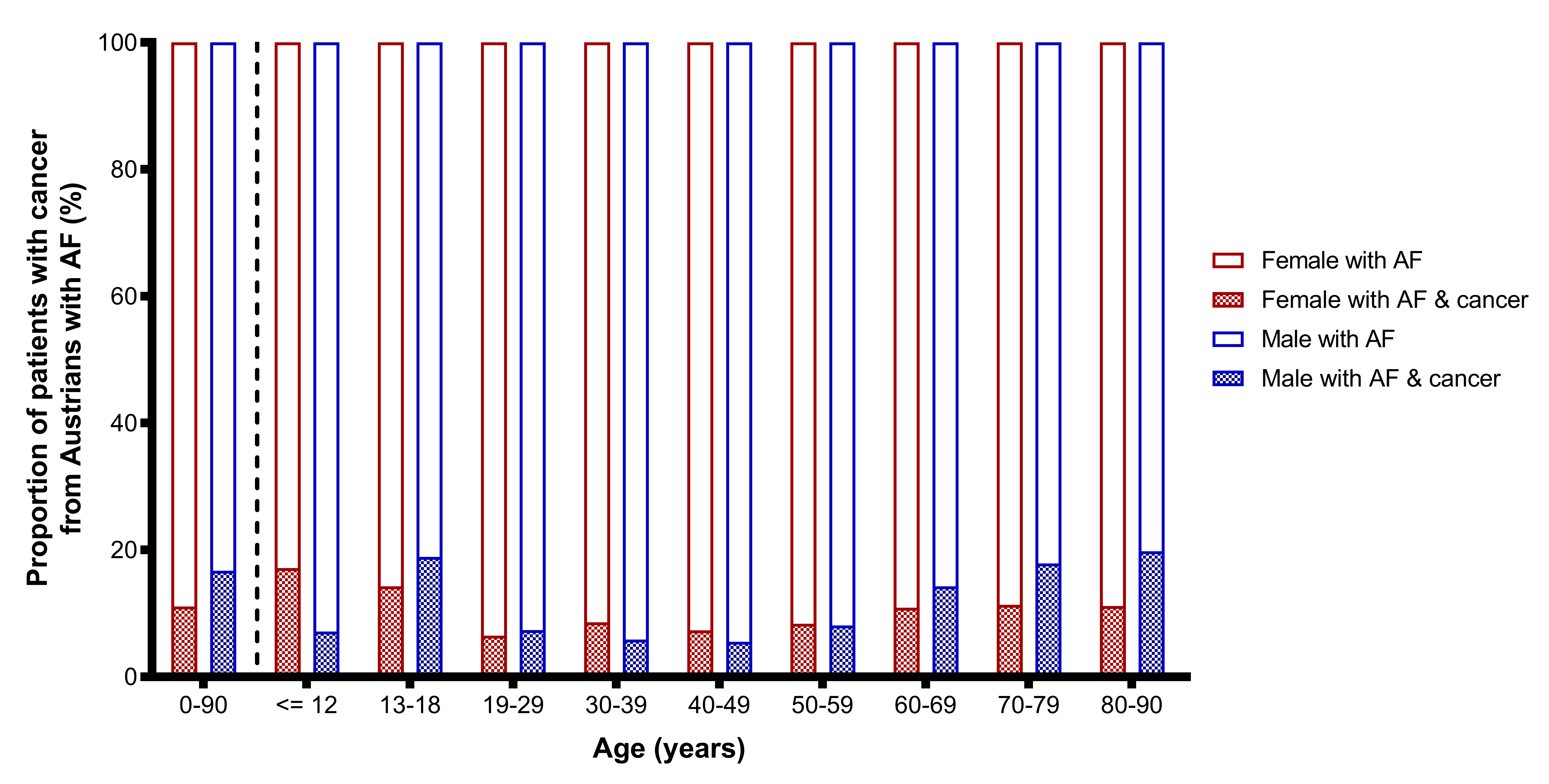

Supplement: Supplementary material [file mmc1.docx]
